# Sexual and procreation preferences of adolescents and young adults infected perinatally with HIV in Southeast Nigeria: a hospital-based cross-sectional study

**DOI:** 10.11604/pamj.2022.42.277.31871

**Published:** 2022-08-12

**Authors:** Kenechukwu Kosisochukwu Iloh, Ijeoma Nnenne Obumneme-Anyim, Chidiebere Donatus Ignatus Osuorah, Ogochukwu Nneka Iloh, Obianuju Ojinika Igbokwe, Cheta Orji-Okafor, Ifeoma Josephine Emodi

**Affiliations:** 1 Department of Paediatrics, College of Medicine University of Nigeria Ituku-Ozalla Campus/University of Nigeria Teaching Hospital Ituku-Ozalla, Enugu, Nigeria,; 2 Child Survival Unit, Medical Research Council UK, The Gambia Unit, Banjul, Gambia,; 3 Nursing Service Department, University of Nigeria Teaching Hospital Ituku-Ozalla, Enugu, Nigeria

**Keywords:** Sexual, procreation, preference, adolescents, perinatal, HIV

## Abstract

Introduction: children with perinatal human immunodeficiency virus (HIV) infection now survive to adolescence and adulthood and so are confronted with issues related to sexuality and sexual reproductive health. This study is aimed at determining the sexual knowledge and behaviours of these adolescents, determining their procreation intention and the impact of their age, gender and understanding of the mother to child transmission risk on their procreation intention.

Methods: a hospital-based cross-sectional study of adolescents and young adults aged 15-24 years infected with HIV in the perinatal period, accessing tertiary care in Enugu. A pretested questionnaire was used to obtain information about socio-demographic variables, sexual knowledge and behaviour, procreation intention and knowledge of prevention of mother-to-child transmission of HIV. Data analysis was descriptive and a test association using fisher´s chi square was done on the variables.

Results: seventy-one adolescents were studied. The majority (95.8%) were less than 20 years of age. Mean age was 17.01 ± 1.80 years with M: F ratio of 1:1.7. Nineteen (26.8%) were sexually active with 15 (78.9%) having single partners. About 80% had their first sexual activities before the age of 18 years. Fifty-six (78.9%) received some form of sex education. Only gender and socio-economic status was significantly related to marriage and procreation intentions of respondents.

Conclusion: there´s need for sustained/intensive education programs and policy on sexual practices with focus on perinatally infected adolescents who may not be well informed on risk and consequences of their sexual preferences.

## Introduction

Children with perinatal Human Immune deficiency virus infection (PHIV) now survive to adolescence and adulthood and so are confronted with issues related to sexuality and sexual reproductive health [1]. The World Health Organization (WHO), reports that approximately 7% of the 36.9 million people living with HIV worldwide in 2014 were infected perinatally [2]. In 2015, approximately 1.8 million adolescents (10-19 years old) were living with HIV in sub-Saharan Africa (SSA), which accounted for 80% of the global HIV infections in adolescents [3].

Adolescents with perinatally acquired HIV infection often learn of their HIV status during this developmental stage when they also have to make decision about initiating sexual relationships, intimate partner notification of their HIV status, managing subsequent rejection by partners, optimal and consistent use of condoms, having children of their own and HIV transmission risk [4, 5]. They are also confronted with other challenges of the adolescent period such as sexuality, alcohol, and drug experimentation which place them at an even higher risk of disease propagation [6]. Delayed pubertal onset is commonly observed among perinatally-infected adolescents, which can result in low self-image, depression, and reproductive consequences. These make them targets for sexual abuse or they may easily engage in sexual intercourse to prove their worth [7, 8].

Little is known about perinatally HIV infected adolescents´ procreation intention and so their sexual and reproductive health needs and sexual rights are often neither recognised nor respected. Understanding sexual health and sexual risk behavior in young people with perinatal HIV is important in order to tailor appropriate education on sexual health, including prevention of unintended pregnancy, acquisition of other sexually transmitted infections and the potential for onward transmission of HIV to partners and offspring [9].

This study is therefore aimed at determining the sexual knowledge and behaviours of these adolescents and young adults, determining their procreation intention and the impact of their age, gender and understanding of the maternal to child transmission (MTCT) risk on their procreation intention.

## Methods

Study setting: this was a hospital-based study carried out at the HIV Clinic of the University of Nigeria Teaching Hospital (UNTH) Enugu, south-east Nigeria.

Study design: a hospital-based cross-sectional study.

Eligibility: all adolescents and young adults aged 15-24 years attending the clinic were eligible if they did not have any of the following exclusion criteria; those that were not aware of their status; those whose mode of infection was not through the mother-to-child transmission (MTCT) route; those whose parents or the study participants declined consent/assent and study participants that were ill to be interviewed during the study period.

Sample size estimation: the sample size was derived from the online Raosoft sample size calculator [10] calculated based on a response rate of 50%, a confidence interval of 99%, and a margin of error of 5%. An ultimate sample size of was 68 calculated.

Study procedure: before the commencement of the study, the questionnaire for the research was pretested in the clinic on five subjects which were not included in the study. This was to check for the appropriateness of the questions and to avoid ambiguity.

The questionnaire was made up of questions eliciting information about socio-demographic variables (age, gender schooling history of subjects, education and occupation of parents/caregiver), sexual knowledge and behaviour (history of sexual activity, age at first sexual exposure, number of partners, history of pregnancy and contraception), procreation intention (desire to marry and have children, HIV status preference of future spouse), and knowledge of prevention of mother-to-child transmission of HIV.

During routine visit to the clinic or club activities from January to September 2019, eligible subjects were consecutively recruited. They were approached individually for consent (if 18 years and above) and parental consent and subject assent for participants younger than 18 years. The purpose of the study was duly explained to the subjects and their parents/guardians in an appropriate language, the consent form was signed or thumb printed. The information on the consent form was translated in the local language for those that could not read or understand English language by the researcher administering the questionnaire.

This was followed by the administration of the questionnaire to the participants by one of the researchers in a comfortable private room to ensure confidentiality. Oyedeji Social Classification System [11] was used to determine the social class of each participant. They were then subdivided into the upper, middle, and lower socio-economic classes. Those in upper social class were represented by classes 1 and 2, 3 for the middle class, while classes 4 and 5 were defined as lower social class. Each score has two variables, occupation, and level of education of both parents/caregivers [11].

Data analysis: data analyses were done using IBM-Statistical Package for Social Sciences (IBM-SPSS) version 20.0. Descriptive statistics were performed and represented using frequency and percentages. Chi square test of association was used to test the level of significance between variables. A p-value of less than 0.05 was regarded as significant.

Ethical consideration: the study was approved by the Health Research and Ethics Committee (HREC) of UNTH. All identifiers were removed from questionnaires. All participants were assured of confidentiality. They were also informed of their rights to opt out at any time during the study period without any form of retribution to them or their relatives.

## Results

**Characteristics of study participants**: seventy-one study participants aged 15-24 years infected with HIV were enrolled into this study. The majority (95.8%) were less than 20 years of age. The mean age of participants was 17.01 ± 1.80 years with a M: F ratio of 1:1.7. Approximately 46.5% and 47.9% of the respondents were from families in the middle and lower socio-economic class respectively. Fifty-five (77.5%) were under the care of their biological parents while the remaining (22.5%) were under the care of non-biological parents. Fifty-seven (80.3%) are currently in school while 14 had dropped out of school. Of the 57 still in school, 49 (86%) were in secondary school while 2 (3.5%) and 6 (10.5%) were in primary and tertiary schools respectively. See Table 1.

**Table 1:** socio-demographic characteristics of participants

S/N	Variable		N (%)
1.	Age (years) Mean ± SD = 17.01 ± 1.80	15 –19	68 (95.8)
		20 –24	3 (4.2)
2.	Gender	Male	26 (36.6)
		Female	45 (63.4)
3.	Social class	Upper	4 (5.6)
		Middle	33 (46.5)
		Lower	34 (47.9)
4.	Caregiver	Parents	55 (77.5)
		Non –parents	16 (22.5)
5.	Occupation status	Dependent	63 (88.7)
		Employed	8 (11.3)
6.	Highest educational attainment	Primary	50 (70.4)
		Secondary	21 (29.6)

Table 2 shows the sexual behavior of study participants. About one third, 19 (26.8%) were sexually active. Of this, 15 (78.9%) had single partners while 4 (21.1%) had multiple partners. The majority (79%) of the participants had their first sexual activities before the age of 18 years with a mean age of first sexual activity being 16.25 ± 2.27 years. Two participants (2.8%) endorsed been coerced into sexual intercourse and both were by peers. Fifty-six (78.9%) had received some form of sex education and almost half (46.4%) received the education from a counselor. Only a quarter, 14 (25.0%) got sex education from either of their parents. Of the nineteen that were sexually active, 6 (31.6%) stated they have been pregnant or impregnated a person whose HIV status was unknown as at the time of their sexual contact. Regarding contraceptive options, 44 (62.0%), 8 (11.3%) and 6 (8.4%) indicated condoms, injectable and oral contraceptive pills only while 13 (18.3%) mentioned more than one method.

**Table 2:** sexual characteristics of participants

S/N	Variable		N (%)
1.	Sexually active?	Yes	19 (26.8)
		No	52 (73.2)
2.	If yes, type of partner	Single	15 (78.9)
		Multiple	4 (21.1)
3.	Age at first sexual exposure (years)	< 18	15 (79.0)
	Mean ± SD = 16.25 ± 2.27	≥ 18	1 (5.2)
4.	Ever been coerced into having sex?	Don't know	3 (15.8)
		Yes	2 (2.8)
		No	69 (97.2)
5.	Ever received any sex education?	Yes	56 (78.9)
		No	15 (21.1)
6.	If yes, by who?	Mother	12 (21.4)
		School teacher	15 (26.8)
		Counselor	26 (46.4)
		Father	2 (3.6)
		Friend	1 (1.8)
7.	Ever been pregnant or impregnated someone?	Yes	6 (31.6)
		No	10 (52.6)
		Refused to mention	3 (15.8)
8.	Type of contraceptives used	Condoms	44 (62.0)
		Injectable	8 (11.3)
		Pills	6 (8.4)
		Multiple methods	13 (18.3)

**Intention for marriage and procreation among respondents:** fifty-six (80%) of respondents were desirous of getting married in the future. Fifty-two (73.2%) would take into consideration the HIV status of their future partner before marriage while 18 (25.4%) do not consider the HIV status of their partner an issue before marriage. However, 43 (60.6%) would prefer their future partner to be HIV negative while 23 (32.4%) would prefer partners who are also HIV positive like themselves. Fifty-seven (80.3%) of the 71 respondents are desirous to have children. Fifty-one (71.8%) mentioned they knew about the prevention of maternal to child transmission of HIV program (PMTCT) and when asked what it was, 31 (60.8%) and 17 (33.3%) correctly and incorrectly described PMTCT. The remaining 3 (5.9%) stated they were not sure what PMTCT entails even though they are aware of it. See Table 3.

**Table 3:** procreation/marriage intention and PMTCT knowledge

S/N	Variable		N (%)
1.	Desire to marry?	Yes	56 (80.0)
		No	9 (12.9)
		Not decided	5 (7.1)
2.	Desire to have children?	Yes	57 (80.3)
		No	3 (4.2)
		I don't know	11 (15.5)
3.	Consideration of HIV status of the man/woman you want to marry?	Yes	52 (73.2)
		No	18 (25.4)
		I don't know	1 (1.4)
4.	Preference of HIV status of partner?	Positive	43 (60.6)
		Negative	23 (32.4)
		Not decided	5 (7.0)
5.	Do you know anyway(s) this can be prevented? (PMTCT awareness)	Yes	51 (71.8)
		No	20 (28.2)
6.	What are those ways? (N = 51) (PMTCT knowledge)	Right	31 (60.8)
		Wrong	17 (33.3)
		Not sure	3 (5.9)

**Socio-demographic determinants of marriage intentions, procreation and knowledge of PMTCT**: Table 4 highlights socio-demographic factors associated with marriage and procreation intentions of respondents. Only gender and socio-economic status was significantly related to marriage and procreation intentions of respondents. Females (28.8%) infected with HIV were more likely to prefer marriage to HIV negative partners than male (6.1%; P=0.031). Likewise, those in the lower (38% for middle and 31% for low) socio-economic classes compared to participants in the high class (4.2%) would put into consideration the HIV status of their future partner before marriage (P=0. 028). Furthermore, HIV positive respondents in the low socio-economic class were significantly more desirous to have children compared to those in the middle and upper socio-economic class (45.1% vs 32.4% vs 2.8%; P=0.019).

**Table 4:** association of procreation/marriage intention to socio-demographic characteristics of respondents

Socio-demographic characteristics		Procreation/marriage intention														
		Desire to marry				Desire to have children				Consideration of partner's HIV status before marriage				What HIV status will you prefer?		
		Yes N (%)	No N (%)	Not decided N (%)	X^2^ (P)	Yes N (%)	No N (%)	I don't know N (%)	X^2^ (P)	Yes N (%)	No N (%)	I don't know N (%)	X^2^ (P)	Pos. N (%)	Neg. N (%)	X^2^ (P)
**Age**	**15 – 19**	53 (75.7)	9 (12.9)	5 (7.1)	0.784 (1.000)	55 (77.5)	3 (4.2)	10 (14.1)	0.848 (0.488)	50 (70.4)	17 (23.9)	1 (1.4)	0.141 (1.000)	41 (62.1)	22 (33.3)	0.003 (1.000)
	**20 – 24**	3 (4.3)	-	-		2 (2.8)	-	1 (1.4)		2 (2.8)	1 (1.4)	-		2 (3.0)	1 (1.5)	
**Gender**	**Male**	18 (25.7)	4 (5.7)	4 (5.7)	4.739 (0.094)	20 (28.2)	1 (1.4)	5 (7.0)	0.442 (0.782)	19 (26.8)	7 (9.9)	-	0.618 (1.000)	20 (30.3)	4 (6.1)	**5.491 (0.031**)
	**Female**	38 (54.3)	5 (7.1)	1 (1.4)		37 (52.1)	2 (2.8)	6 (8.5)		33 (46.5)	11 (15.5)	1 (1.4)		23 (34.8)	19 (28.8)	
**Social class**	**Upper**	2 (2.9)	-	1 (1.4)	4.483 0.378)	2 (2.8)	-	2 (2.8)	**10.981 (0.019)**	3 (4.2)	-	1 (1.4)	**20.430 (0.028)**	1 (1.5)	3 (4.5)	5.581 (0.052)
	**Middle**	26 (37.1)	4 (5.7)	3 (4.3)		23 (32.4)	3 (4.2)	7 (9.9)		27 (38.0)	6 (8.5)	-		24 (36.4)	7 (10.6)	
	**Lower**	28 (40.0)	5 (7.1)	1 (1.4)		32 (45.1)	-	2 (2.8)		22 (31.0)	12 (16.9)	-		18 (27.3)	13 (19.7)	
**Caregiver**	**Parents**	42 (60.0)	7 (10.0)	5 (7.1)	1.629 (0.663)	41 (57.7)	3 (4.2)	11 (15.5)	5.073 (0.065)	41 (57.7)	13 (18.3)	1 (1.4)	0.631 (0.641)	36 (54.5)	16 (24.2)	1.797 (0.215)
	**Non – parents**	14 (20.0)	2 (2.9)	-		16 (22.5)	-	-		11 (15.5)	5 (7.0)	-		7 (10.6)	7 (10.6)	
**Highest educational attainment**	**Primary**	37 (52.9)	8 (11.4)	5 (7.1)	4.132 (0.187)	41 (57.7)	1 (1.4)	8 (11.3)	2.071 (0.378)	35 (49.3)	15 (21.1)	-	4.064 (0.121)	27 (40.9)	18 (27.3)	1.653 (0.270)
	**Secondary**	19 (27.1)	1 (1.4)	-		16 (22.5)	2 (2.8)	3 (4.2)		17 (23.9)	3 (4.2)	1 (1.4)		16 (24.2)	5 (7.6)	

X^2^ = Chi – square value; Bold values of P significant at < 0.05

More respondents whose primary caregiver were their parents were significantly more aware of the prevention of maternal to child transmission of HIV program compared to those who primary caregiver were non-parents (50.7% vs. 21.1%; P=0.029). No other socio-demographic factors considered in this study was significantly associated with awareness of maternal to child transmission of HIV (MTCT) and prevention programs to mitigate HIV transmission to newborn (PMTCT) Table 5.

**Table 5:** association of MTCT awareness, knowledge and perception to socio-demographic characteristics of respondents

Socio-demographic characteristics		MTCT Awareness, knowledge and perception											
		MTCT awareness			MTCT aware and still want to marry and have children			PMTCT awareness			PMTCT knowledge		
		Yes	No	X^2^ (P)	Yes	No	X^2^ (P)	Yes	No	X^2^ (P)	Right	Wrong	X^2^ (P)
**Age**	**15 – 19**	62 (87.3)	6 (8.5)	0.289 (1.000)	58 (82.9)	9 (12.9)	0.929 (1.000)	50 (70.4)	18 (25.4)	2.294 (0.554)	31 (64.6)	17 (35.4)	Not computable
	**20 – 24**	3 (4.2)	-		2 (2.9)	1 (1.4)		1 (1.4)	2 (2.8)		-	-	
**Gender**	**Male**	24 (33.8)	2 (2.8)	0.030 (1.000)	20 (28.6)	6 (8.6)	2.611 (0.158)	16 (22.5)	10 (14.1)	2.148 (0.176)	6 (12.5)	8 (16.7)	4.079 (0.095)
	**Female**	41 (57.7)	4 (5.6)		40 (57.1)	4 (5.7)		35 (49.3)	10 (14.1)		25 (52.1)	9 (18.8)	
**Social class**	**Upper**	4 (5.6)	-	1.234 (0.601)	3 (4.3)	-	1.086 (0.695)	4 (5.6)	-	4.589 (0.146)	2 (4.2)	2 (4.2)	0.498 (0.819)
	**Middle**	29 (40.8)	4 (5.6)		27 (38.6)	6 (8.6)		20 (28.2)	13 (18.3)		13 (27.1)	6 (12.5)	
	**Lower**	32 (45.1)	2 (2.8)		30 (42.9)	4 (5.7)		27 (38.0)	7 (9.9)		16 (33.3)	9 (18.8)	
**Caregiver**	**Parents**	51 (71.8)	4 (5.6)	0.438 (0.611)	46 (65.7)	8 (11.4)	0.054 (1.000)	36 (50.7)	19 (26.8)	**4.904 (0.029)**	20 (41.7)	15 (31.3)	3.128 (0.099)
	**Non – parents**	14 (19.7)	2 (2.8)		14 (20.0)	2 (2.9)		15 (21.1)	1 (1.4)		11 (22.9)	2 (4.2)	
**Highest educational attainment**	**Primary**	47 (66.2)	3 (4.2)	1.312 (0.352)	43 (61.4)	6 (8.6)	0.556 (0.473)	39 (54.9)	11 (15.5)	3.179 (0.089)	25 (52.1)	13 (27.1)	0.116 (0.727)
	**Secondary**	18 (25.4)	3 (4.2)		17 (24.3)	4 (5.7)		12 (16.9)	9 (12.7)		6 (12.5)	4 (8.3)	

X^2^ = Chi – square value; Bold values of P are statistically significant at p < 0.05

## Discussion

This study was conducted to determine the sexual behaviors and procreation intention of adolescents and young adults aged 15-24 years who were perinatally infected by HIV (PHIV+). The mean age of first sexual encounter in this study was at 16 years. This agreed with Kenu *et al*. [12] in Ghana and Mbalinda *et al*. [13] in Uganda, but was two years later than the age documented by other researchers in Nigeria and outside Nigeria. In a study in Jos by Yiltok *et al*. [14] the age at first sexual activity was 14 years. Kaushik *et al*. [15] in another comparative study among adolescents in New Jersey USA, reported age at initiation of sexual activity for PHIV+ adolescents at 14 years (IQR of 13-16) and 14 years (IQR of 12-15) for HIV-uninfected adolescents. Another study by Kennedy *et al*. [16] in post conflict Liberia showed that over a third of surveyed adolescent had first sex at ages 14 or younger, and 66% of first sexual encounters were unprotected.

Our study also reported that about a quarter of the PHIV+ individuals were sexually active. This is similar to the documentations of Adu-Mireku [17] in Ghana but different from the findings of researchers [15] in New Jersey in the US where 58% of adolescents living with HIV were found to be sexually active. This higher proportion of sexually active adolescent in the USA may be a reflection of their very liberal society compared to some African society where cultural beliefs and traditions frown at sexual activities outside the confines of marriage. Unlike the referenced study in New Jersey, majority of the adolescents in our study who were sexually active had single sexual partners. This is a welcome development as having multiple sexual partners is a reflection of the daring, experimentation and risk-taking behavior of the adolescent which is also a major risk factor in HIV propagation.

Although about 80% of the study participants had received some form of sex education, this did not significantly impact on their sexual behavior as about 1 in 5 participants who were sexually active, had multiple sexual partners and a third had reported having been pregnant. Ezeanolue *et al*. [18] in a survey of adolescents and young adults (13-24 years) also reported a similar trend where it was noted that even though as much as 89% of their study population had received sex education, almost 20% of the females had been pregnant. Furthermore, it was noted in our study that almost half of the study participants received sex education from healthcare workers. This finding was corroborated by Kenu *et al*. [12] in Ghana where more participants received sex education from health workers compared to parents/guardians (33.7% vs 26.3%). The higher proportion of adolescents receiving sex education from counsellors is evidence that some parents and guardians still experience discomfort sometimes in discussing sexuality with their children. Some of them perceive this as encouraging sexual behaviour and experimentation among adolescents [19, 20]. Another study by Elkington *et al*. [21] in 2010 also identified barriers such as cultural norms and social values. As a result, most of these adolescents indicated that they feel more comfortable talking about sex education to their counsellors (also referred to as health educators) at the HIV clinic and therefore tend to open up to them. The importance of early sex education for the adolescents cannot be over emphasized. Families and healthcare workers both have crucial roles to play in early commencement of sex education in order to help these children understand the importance of healthy sexual and reproductive health practices.

Like most people in the general population, our study noted that PHIV+ were desirous of marriage and procreation. More than half of surveyed adolescents expressed intention of getting married to partners that are HIV negative with considerably more females and individuals from low socio-economic class expressing this choice. Regardless of choice, there is documented clinical evidence that even HIV positive individuals can be re-infected with more viral load from the same strain and/or superinfected with a different strain of the HIV. Both scenarios can complicate treatment and control of their index infections [22, 23]. It is therefore expedient to create awareness of the risk of further HIV infections in partners regardless of their HIV status. This further emphasizes the need for education on safe sexual behaviors and practices among people living with HIV.

A large number of the study participants (91.5%) were aware of the PMTCT program, even though only 60.8% could correctly describe it and a significant number of those participants who were aware of the PMTCT were those whose primary caregivers were the parents. This is not surprising as it was their mode of acquiring the HIV and hence suggests early disclosure on the part of the parents of both the HIV status of the child and the route of acquisition. The benefits of this disclosure thus manifest in the adolescent being adequately informed of PMTCT and hence the further spread of HIV to the younger generation can be somewhat reduced.

Limitations: the main limitation of this study is that it was a hospital based cross-sectional study. Also, because of our focus on adolescent infected perinatally with HIV, a further limitation is the small sample size enrolled which may impact on the power and generalizability of study result. It is therefore recommended that the findings of this study be interpreted in light of these limitations.

## Conclusion

The findings of this study demonstrate the need for a sustained and more intensive education programs and policy on sexual practices and public health safety for people living with HIV.

### What is known about this topic

- Adolescents with perinatal HIV infection are sexually active;
- There are gaps in sexual knowledge among these HIV infected adolescents.

### What this study adds

- To the best of our knowledge, this is the first study of its kind in West Africa and so adds the perspective of West African adolescents to the body of knowledge;
- Most of the adolescents had received sex education and half of them were from counselors.
